# Long-term tracking of neurological complications of encephalopathy and myopathy in a patient with nephropathic cystinosis: a case report and review of the literature

**DOI:** 10.1186/1752-1947-2-235

**Published:** 2008-07-18

**Authors:** Marcus Müller, Andrea Baumeier, EB Ringelstein, IW Husstedt

**Affiliations:** 1 Department of Neurology, Universitätsklinikum Münster, Albert-Schweitzer-Strasse, D-48129 Münster, Germany

## Abstract

**Introduction:**

Cystinosis is a hereditary storage disease resulting in intracellular accumulation of cystine and crystal formation that causes deterioration of the function of many organs. The major clinical symptom is renal failure, which progresses and necessitates renal transplantation at the beginning of the second decade of life. Encephalopathy and distal myopathy are important neurological long-term complications with a major impact on the quality of life of these patients. Application of cysteamine is the only specific therapy available; it decreases the intracellular cystine level and delays or may even prevent the failure of organ functions.

**Case presentation:**

We present the case of a 38-year-old woman with cystinosis and the long-term tracking of her neurological symptoms under cysteamine treatment.

**Conclusion:**

This case report describes a long observation period of neurological complications in a person with cystinosis who had strikingly different courses of encephalopathy and myopathy while on cysteamine treatment. Although encephalopathy was initially suspected, this did not develop, but distal myopathy progressed continuously despite specific therapy.

## Introduction

Nephropathic cystinosis is a rare, autosomal recessive disease, based on a defect of the carrier-mediated transport of cystine across the lysosomal membrane. As a consequence cystine accumulates and forms crystals in most tissues, which causes deterioration of organ function to different degrees [1].

The disease manifests with developmental retardation, polyuria, proteinuria and glucosuria. Without specific treatment a renal tubular Fanconi syndrome and consequently terminal renal failure occur. Since the life expectancy of patients with cystinosis has increased in recent times because of the availability of renal transplantation and a specific therapy, complications of other organ dysfunctions can develop.

From a neurological perspective, important long-term complications of nephropathic cystinosis are encephalopathy and distal myopathy.

In 1987 Jonas et al. described for the first time cystine accumulation in the brain of a patient with cystinosis who suffered from confusion and memory loss. Cranial computed tomography (CCT) confirmed cerebral atrophy [2]. Subsequently, neurological symptoms such as mental retardation, epileptic seizures, tremor and pyramidal syndromes were observed in patients with cystinosis [3,4].

In parallel, cystine accumulation was detected in muscular tissue and in 1988 the first case of severe myopathy was reported [5]. A 20-year-old patient with cystinosis was described, suffering from progressing dysphagia and weakness of the hands and arms. Subsequently the patient lost the ability to sit up without assistance and could walk only three blocks without resting. Post-mortem muscle studies revealed peri- and endomysial cystine crystals. Shortly afterwards, an *in vitro *study confirmed cystine accumulation in myotube cells [6] from patients with cystinosis.

A study of 54 post-transplant patients with cystinosis found distal myopathy in 13 of these patients [7]. Clinical signs consisted of weakness and atrophy of the small muscles of the hand, dysphagia and facial weakness. Nerve conduction studies revealed no abnormalities. Dysphagia and swallowing dysfunction were evaluated in a separate study by Sonies et al. [8], who demonstrated age-dependent dysphagia in nearly all patients with cystinosis. In addition Vester et al. demonstrated electrophysiological signs of myopathy in patients with cystinosis without obvious weakness [9]. Myopathic changes were most prominent in the small muscles of the hand.

The only specific therapy for cystinosis is the oral application of cysteamine (cysteamine bitartrate, Cystagon^®^), which transforms cystine into a soluble form that may leave the cell [10]. A beneficial effect for cystinosis-associated encephalopathy is described. Whether or not cysteamine therapy influences cystinosis-associated myopathy [3] is uncertain, however a positive effect on dysphagia as a myopathic symptom has been suggested [11].

## Case presentation

We report the clinical course of a 38-year-old woman, who had been entirely normal at birth, but by the age of 6 months had developed polydipsia, polyuria and vomiting, which led to the diagnosis of nephropathic cystinosis.

By the age of 13 years, the patient suffered from terminal renal insufficiency and finally required dialysis. One year later she underwent renal transplantation.

At the age of 23 years, the patient noticed a lack of concentration and motor coordination. She underwent neurological examination by a neuropediatrician experienced with cystinosis-associated symptoms. No clinical or neurophysiological disturbances were identified; in particular, no clinical signs of cerebellar dysfunction, dysmetria in point-to-point movements, dysdiadochokinesis, ataxia or dysarthria were detectable.

**Figure 1:**
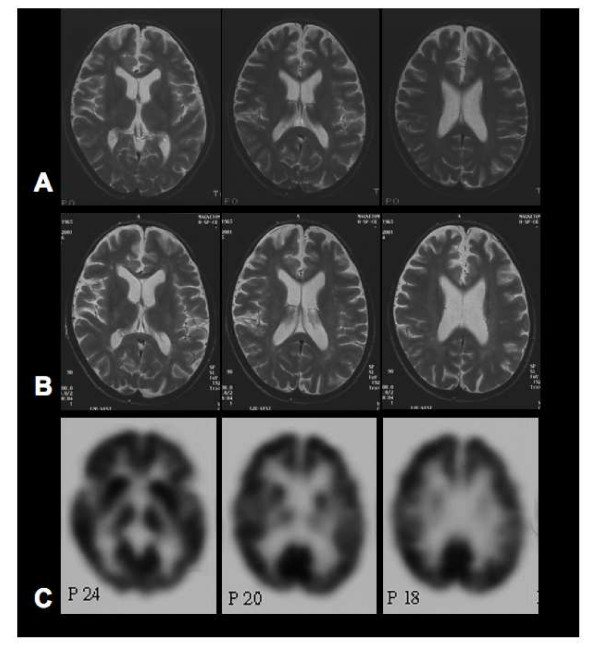
**Imaging studies**. (A) Initial magnetic resonance imaging revealed signs of cerebral atrophy with a prominent inter-hemispheral fissure at the age of 23 years. (B) The second magnetic resonance imaging 11 years later did not reveal any progression of cerebral atrophy nor any other signs of cystinosis-associated encephalopathy. (C) Positron emission tomography at the age of 34 years demonstrated normal cortical glucose utilisation without signs of encephalopathy.

Two years later, due to symptom persistence, the patient had a second neurological examination. Asymmetric tendon reflexes, slightly dysmetric point-to-point movements and a minimal dysdiadochokinesis pointing towards minor cerebellar dysfunction were observed at that point. Electrophysiology revealed a pathological P300 potential. Signs of myopathy were not observed. An initial magnetic resonance tomography (MRT) scan revealed cerebral atrophy (Figure 1A).

By the age of 29 years, intermittent episodes of somnolence were reported and the patient was admitted to the neurology ward. A CCT scan revealed generalised cortical atrophy. Physical examination demonstrated asymmetric tendon reflexes and no signs of myopathy but there was dysdiadochokinesis, dysmetric point-to-point movements and a gait imbalance when walking in a straight line. At this point specific therapy with Cystagon^® ^was commenced (30 mg/kg, three daily doses with the highest dosage in the evening) and the effect of the therapy was controlled by determining the lymphocyte cysteine levels (between 0.2 and 0.47 nmol/mg protein). Higher dosages were not tolerated by the patient.

By the age of 32 years, myopathic signs were observed despite effective cysteamine therapy, and cystinosis-associated distal myopathy was diagnosed by clinical criteria. During the following 2 years, the patient suffered from recurrent depressive episodes according to ICD-10 (F33.2). Due to additional difficulties in concentration progressive cystinosis-associated encephalopathy was suspected and the patient was again admitted to the neurology ward.

She was admitted at the age of 34 years and reported diffuse complaints such as holocephalic headache, generalised weakness and difficulties in swallowing. She stated that for the last 3 months she had spent most of the daytime in bed and had felt very listless. The neurological examination after admission revealed no clinical signs of encephalopathy. Major cognitive or amnestic deficits were not observable. Tendon reflexes were symmetrical and pathologic pyramidal signs were not present. Furthermore, the clinical examination did not reveal any signs of the cerebellar dysfunction that had been documented 5 years earlier. Strength testing demonstrated symmetrical distal paresis of the arms and legs which was most pronounced in the hands and feet. Small muscles of the feet and hands were atrophic. The mini mental state examination (MMSE) was normal.

In contrast to the suspected cystinosis-associated encephalopathy, the patient's interview again revealed signs of a recurrent depressive episode. She reported depressed mood, anhedonia, loss of interest and enjoyment, and reduced energy, combined with low self-esteem and a sense of worthlessness and despair. To examine the impairment of other organ functions by the cystinosis, an extensive set of laboratory tests was performed. Glucose and HbA1C testing ruled out diabetes mellitus but an oral glucose tolerance test (OGTT) confirmed impaired glucose tolerance. Thyroid-stimulating hormone (TSH) level was normal under treatment with 100 μg L-thyroxin, which had been commenced because of cystinosis-associated hypothyroidism. Examination of the eyes revealed opacity of the corneas and decreased vision despite the regular use of cysteamine eyedrops.

To exclude cystinosis-associated encephalopathy, diagnostic procedures were performed including a second MRT (Figure 1B), electroencephalography (EEG), a [18F]-2-fluoro-deoxy-D-glucose positron emission tomography (FDG-PET, Figure 1C) and a neuropsychological examination. The MRT scan revealed brain atrophy but compared with the previous scan showed no progression. EEG was normal and the FDG-PET results showed normal brain metabolism without any further focal or general metabolic disturbances. Extensive neuropsychological testing revealed distractibility, poor concentration and memory impairments consistent with a depressive episode. Higher cortical functions were intact.

Concerning the clinical suspicion of cystinosis-associated myopathy, electromyography (EMG) and nerve conduction studies revealed a typical myopathic EMG pattern with spontaneous activity in all muscles examined. Muscle potentials were shortened with low amplitude. Motor-unit recruitment was abnormally early. Myopathic changes were most pronounced in the small musculature of the hands. Nerve conduction studies were normal. The patient refused further evaluation by muscle biopsy.

A depressive episode was diagnosed by an experienced psychiatrist according to ICD-10 (F33.2) criteria and antidepressive therapy with a selective serotonin re-uptake inhibitor (SSRI) was started. The diagnosis of cystinosis-associated myopathy was confirmed and cystinosis-associated encephalopathy ruled out.

After discharge from hospital, the patient was regularly examined in the outpatient clinic. One year after admission, no clinical signs of progressive encephalopathy and no symptoms of recurring depression were observed. She reported vitality and days spent in planning and enjoying activities, although muscle wasting progressed. In the meantime, corneal transplantations were successfully performed and resulted in a dramatic improvement in vision. She reported that motor functions involved in, for example, cutting up meat or climbing stairs were increasingly difficult. EMG confirmed progress of the myopathy with increased spontaneous activity and short and polyphasic motor unit potentials, mainly in distal arm muscles. Acoustic and somatosensory evoked potentials and EEG were normal. The neuropsychological examination was repeated and in parallel with improvement of depressive symptoms the previous cognitive deficits were improved. Higher cortical functions were again unimpaired.

One year later, at the age of 38 years, the patient was examined again and no major changes of cognitive functions and mood were observed. Compared with the previous examination, the myopathy had progressed and the patient now reported that, in addition to the previously reported motor symptoms, writing and chewing were more difficult. The cysteamine therapy was continued without disruption.

## Discussion

Encephalopathy and myopathy are long-term complications of nephropathic cystinosis. These complications are become increasingly apparent because the lifespan of patients with cystinosis is increasing. As a result of renal transplantation and the availability of cysteamine, patients with cystinosis who are older than 20 years are no longer uncommon and the number of adults with cystinosis will increase in the future.

Cystinosis-associated encephalopathy was first described in 1982 in a 19-year-old patient with cystinosis who had hemiparesis and dysarthria [12]. In the following years many studies reported symptoms of encephalopathy in patients with cystinosis and confirmed this first observation. It was estimated that 50% of all untreated patients with cystinosis have signs of encephalopathy [4]. A follow-up study of treated patients found a lower incidence of neurological complications [13].

Cystinosis-associated encephalopathy may occur with different manifestations. Cerebral atrophy was observed in several studies and although terminal renal insufficiency could also lead to cerebral atrophy, the frequency and severity of brain atrophy in untreated patients with cystinosis is higher than in patients with terminal renal insufficiency alone. In addition, hydrocephalus malresorptivus, focal demyelinated lesions and cystic necrosis were observed. Some patients suffer from recurrent phases of somnolence, epileptic seizures, pyramidal tract lesions and mental retardation. Broyer et al. [4] analysed a series of cases and found that patients with cystinosis who are older than 23 years have an increasing risk for cystinosis-associated encephalopathy. Half of the patients had clinical signs of encephalopathy. In addition it was demonstrated that subsequent therapy with cysteamine could stop the progression of encephalopathy and in some cases could improve other neurological deficits [4].

Muscular tissue accumulates cystine as well and the first case of cystinosis-associated myopathy was described in 1988 [5]. Clinically, patients develop a distally pronounced atrophic myopathy with difficulties in swallowing, which is a pattern comparable to other myopathies such as inclusion body myositis. In our patient cystinosis-associated myopathy was first diagnosed in 1997. The myopathy must have developed between 1994 and 1997 because the neurological examination in 1994 revealed no obvious signs of myopathy. In 2000, muscular weakness and distal atrophies were the distinct pathologic features and progressed continuously despite cysteamine therapy over the following years.

Cysteamine is the only effective therapy for cystinosis. It has been available since 1976 [14,15] and was approved for the therapy of cystinosis in 1994. Cysteamine transforms intralysosomal cystine to cysteine, which is able to leave the cell independently of the defective cystine transporter. Measuring the cystine level of lymphocytes or fibroblasts can monitor the therapeutic effect. With early and subsequent cysteamine therapy renal function can be preserved. In addition, a beneficial effect on cystinosis-associated encephalopathy is described [4], whereas the effect of cysteamine on cystinosis-associated myopathy has not yet been proved, although the beneficial effect on dysphagia suggests such an effect.

A beneficial effect of the therapy can also be assumed in the case reported here. At the age of 25 years, before the initiation of cysteamine therapy, the patient had signs of cerebral atrophy confirmed by an MRT scan. Cerebral atrophy in a patient with cystinosis points towards a developing encephalopathy. In contrast, with specific cysteamine treatment no progression of brain atrophy could be observed in the following years. The MRT scan carried out 11 years after the first examination revealed unaltered cerebral atrophy without further structural abnormalities. In addition, no clinical signs of cystinosis-associated encephalopathy were observed at that time.

Considering that the study by Broyer et al. demonstrated that the percentage of encephalopathy-free patients with cystinosis according to age declines from 95% at the age of 23 years to 55% at the age of 27 years, the course of this patient, who is now 39 years old and without signs of encephalopathy, is remarkable.

In contrast, cystinosis-associated myopathy, diagnosed in 1997, became clinically obvious during cysteamine therapy and progressed despite this therapy. Intracellular cystine was regularly determined in lymphocytes and proved the efficacy of the therapy. Myopathic changes were mostly prominent in distal limb muscles and led to restrictions in motor function of the hands, which is the typical course of cystinosis-associated myopathy.

Although no study has yet addressed the question of whether cysteamine is an effective treatment for cystinosis-associated myopathy, Gahl et al. demonstrated that cysteamine is able to deplete muscular tissue of cystine. In our patient, the myopathy progressed, pointing towards an insufficient effect of the cysteamine therapy to prevent or stop progression of the cystinosis-associated myopathy. Several reasons could explain this observation: one could speculate that muscular tissue is more susceptible to intracellular cystine accumulation and a cystine concentration that will not affect non-muscular tissue could be harmful to muscular tissue. Considering the anatomical structure and the mechanical stress which muscular tissue undergoes, it is conceivable that muscular tissue might be more susceptible to cystine-mediated damage. Referring to the distribution pattern of the myopathy, one could speculate that the cystine load of different muscles and the associated muscle damage are also variable and normal cystine levels in some muscles may not exclude high levels in others. Another explanation could be that the effect of cysteamine is variable in different types of muscle.

Although the above explanations are speculative, this case report highlights the important need for a better understanding of the pathogenesis of the different cystinosis complications, which can have different dynamics.

## Conclusion

In conclusion, we have presented and discussed the case a 38-year-old patient with cystinosis. The course of long-term neurological complications of encephalopathy and myopathy were observed over a period of more than 15 years. Effective cysteamine therapy could not prevent the progression of cystinosis-associated myopathy, whereas early signs of developing encephalopathy did not progress over almost 15 years. This case report demonstrates the possible divergent course of cystinosis-induced neurological complications and the need for new therapeutic options.

As the lifespan of patients with cystinosis has increased over the last decades, the neurological complications of this disease will increasingly challenge their physicians.

## Abbreviations

CCT, cranial computed tomography; EEG, electroencephalography; EMG, electromyography; FDG-PET, [18F]-2-fluoro-deoxy-D-glucose positron emission tomography; MRT, magnetic resonance tomography; OGTT, oral glucose tolerance test; SSRI, selective serotonin re-uptake inhibitor; TSH, thyroid-stimulating hormone.

## Competing interests

The authors declare that they have no competing interests.

## Consent

Written informed consent was obtained from the patient for publication of this case report and any accompanying images. A copy of the written consent is available for review by the Editor-in-Chief of this journal.

## Authors' contributions

MM examined the patient, reviewed the literature, wrote the manuscript and prepared the Figure. AB examined the patient and prepared the patient's history. ER and IH critically reviewed the manuscript and contributed to the discussion.
